# Editorial: Healthcare in the age of sapient machines: physician decision-making autonomy faced with artificial intelligence. Ethical, deontological and compensatory aspects

**DOI:** 10.3389/fmed.2024.1477371

**Published:** 2024-08-28

**Authors:** Filippo Gibelli, Giovanni Maio, Giovanna Ricci

**Affiliations:** ^1^Section of Legal Medicine, School of Law, University of Camerino, Camerino, Italy; ^2^Department of Medical Ethics and the History of Medicine, University of Freiburg, Freiburg, Germany

**Keywords:** artificial intelligence, decision support models, decision-making, automation, bioethics

The decision to explore this topic was inspired by the growing recognition that artificial intelligence (AI) is assuming an increasingly significant role in medical practice. In recent years, in fact, AI has entered the healthcare sector in a significant way, thanks to the extraordinary technological developments that have enabled the transition from traditional AI systems, such as artificial neural networks, rule-based algorithms, expert systems, and knowledge-based artificial intelligence, to advanced AI systems, such as machine learning and deep learning. The use of these systems in healthcare, which are capable of operating with a high degree of autonomy, represents an invaluable resource in terms of the quality of care provided, but poses obvious ethical and deontological problems. In particular, the introduction of highly automated AI raises crucial questions concerning the decision-making autonomy of physicians, a crucial element in guaranteeing the quality of care and trust in the doctor-patient relationship.

The aim of this Research Topic was to investigate the state of the art regarding the relationship between physicians' decision-making autonomy and the use of highly automated AI systems in healthcare. We wanted to analyze the critical issues emerging in routine medical practice, examine the attempts implemented to solve the problem and assess the importance of Research Topic for healthcare professionals and patients. Through the six articles presented, we attempted to provide a comprehensive overview of the topic as well as to stimulate a constructive discussion on these key issues for the future of healthcare.

The Research Topic features six articles, comprising 1 mini-review, 3 reviews, 1 “hypothesis and theory” article and 1 perspective article. All contributions provided valuable elements to clarify the physiognomy of human-machine interaction and to characterize the profiles of decision-making autonomy of the contemporary age physician.

The mini review by Terranova et al. discusses the impact of AI on professional liability in healthcare. It highlights the potential of AI to enhance patient safety and improve healthcare outcomes but also raises ethical and legal concerns. The review examines how AI can assist in evaluating malpractice claims by analyzing factors such as informed consent and adherence to standards of care. It emphasizes the need for new legislative regulations and specialized expert witnesses to address AI's integration into legal medicine. The review concludes that combining AI with human judgment can improve liability assessments, but a cautious approach is necessary to avoid complete automation.

The review by Cestonaro et al. examines the use of AI in medical diagnostics, highlighting its potential to improve diagnostic accuracy and reduce clinician workload. However, it also addresses significant ethical and legal challenges, including biases in AI algorithms, privacy concerns, and the “black box” phenomenon, where AI decision-making is not transparent. The review emphasizes the inadequacy of current legal frameworks to address AI-related errors, advocating for clear regulatory guidelines to define liability and ensure patient safety. It concludes that legal reforms are necessary to navigate the complexities of AI in healthcare effectively.

The review by Saccà et al. examines the integration of AI in healthcare and its implications for patient consent. It highlights how AI is revolutionizing clinical practice, the patient-caregiver relationship, and the diagnostic and treatment processes. The review identifies the main ethical and legal challenges posed by AI, especially regarding informed consent and patient autonomy. It provides an overview of current guidelines and legislation on AI use in public health and proposes seven key principles to ensure informed patient consent in the AI era. These principles aim to balance technological advancements with the patient's right to self-determination and health.

The review by Sablone et al. examines the ethical and medico-legal implications of AI in healthcare from an Italian perspective. It highlights the benefits of AI, such as enhancing diagnostic accuracy and treatment efficacy, but also addresses concerns about the reduction in doctors' decision-making autonomy and the opacity of AI decision processes (the “black box” issue). The article also discusses the need for new legal frameworks to manage liability in cases of AI-related medical errors and emphasizes the importance of integrating AI ethically and transparently into healthcare systems while maintaining human oversight to ensure patient safety and trust.

The “hypothesis and theory” article by Funer and Wiesing explores the ethical implications of AI support tools in medical practice, focusing on the physician's autonomy. The authors argue that physician autonomy, essential for patient wellbeing, must be maintained even with AI integration. They identify three conditions for this: adequate information about AI tools, physician competence to integrate AI into decision-making, and a voluntary context allowing deviation from AI recommendations. The article emphasizes that AI should support, not replace, human decision-making and calls for a design and implementation of AI that upholds the physician's professional autonomy.

The perspective article by Li et al. discusses the integration of AI in traditional Chinese medicine (TCM), highlighting both opportunities and challenges. AI can enhance diagnostic accuracy, personalize treatments, and facilitate data-driven insights, benefiting TCM practice. However, AI cannot replace the humanistic aspects of TCM, such as understanding patients' emotions and providing empathetic care. The article also addresses the difficulty in establishing trust between AI and practitioners, the incomplete legislation on AI-assisted TCM, and the need for preserving the human element in patient care while leveraging AI technology for improved outcomes.

In conclusion, the Research Topic allowed for a comprehensive exploration of the most relevant medico-legal and bioethical implications of the use of highly automated artificial intelligence systems in medicine, including the issue of the preservation of the physician's decision-making autonomy. The articles collectively highlight the dual potential of AI: enhancing diagnostic accuracy, patient safety, and healthcare outcomes, while simultaneously posing significant ethical, legal, and deontological challenges. Key themes include the necessity for new legislative frameworks to address AI-related liabilities, the importance of maintaining physician autonomy and informed consent, and the imperative to balance AI's technological advancements with the human elements of patient care. The Research Topic underscores the critical need for cautious integration of AI in healthcare, advocating for a symbiotic relationship where AI supports but does not replace human judgment. This approach aims to preserve the trust and quality inherent in the doctor-patient relationship, ensuring that AI advancements contribute positively to the future of healthcare.

